# Community-Engaged Use of Low-Cost Sensors to Assess the Spatial Distribution of PM_2.5_ Concentrations across Disadvantaged Communities: Results from a Pilot Study in Santa Ana, CA

**DOI:** 10.3390/atmos13020304

**Published:** 2022-02-11

**Authors:** Shahir Masri, Kathryn Cox, Leonel Flores, Jose Rea, Jun Wu

**Affiliations:** 1 Department of Environmental and Occupational Health, Program in Public Health, University of California, Irvine, CA 92697, USA; 2 Madison Park Neighborhood Association, GREEN-MPNA Programs, Santa Ana, CA 92707, USA; 3 Department of Anthropology, School of Social Sciences, University of California, Irvine, CA 92697, USA

**Keywords:** air pollution, environmental justice, citizen science, community, PM_2.5_

## Abstract

PM_2.5_ is an air pollutant that is widely associated with adverse health effects, and which tends to be disproportionately located near low-income communities and communities of color. We applied a community-engaged research approach to assess the distribution of PM_2.5_ concentrations in the context of community concerns and urban features within and around the city of Santa Ana, CA. Approximately 183 h of one-minute average PM_2.5_ measurements, along with high-resolution geographic coordinate measurements, were collected by volunteer community participants using roughly two dozen low-cost AtmoTube Pro air pollution sensors paired with real-time GPS tracking devices. PM_2.5_ varied by region, time of day, and month. In general, concentrations were higher near the city’s industrial corridor, which is an area of concern to local community members. While the freeway systems were shown to correlate with some degree of elevated air pollution, two of four sampling days demonstrated little to no visible association with freeway traffic. Concentrations tended to be higher within socioeconomically disadvantaged communities compared to other areas. This pilot study demonstrates the utility of using low-cost air pollution sensors for the application of community-engaged study designs that leverage community knowledge, enable high-density air monitoring, and facilitate greater health-related awareness, education, and empowerment among communities. The mobile air-monitoring approach used in this study, and its application to characterize the ambient air quality within a defined geographic region, is in contrast to other community-engaged studies, which employ fixed-site monitoring and/or focus on personal exposure. The findings from this study underscore the existence of environmental health inequities that persist in urban areas today, which can help to inform policy decisions related to health equity, future urban planning, and community access to resources.

## 1. Introduction

Air pollution is a widely recognized public health threat known to be associated with a range of adverse health outcomes, including asthma, cardiovascular disease, and respiratory disease, as well as all-cause mortality and hospital admissions [1–5]. Studies have also found air pollution to exacerbate viral infections such as COVID-19, among other disease, as well as impact mental health [6–8]. In a recent study examining “urban-associated diseases”, such as asthma, allergies, and cancer, air pollution was found to be the characteristic of cities that was most frequently associated with negative health effects [9].

Particulate matter with an aerodynamic diameter of less than 2.5 μm (PM_2.5_) is particularly detrimental to health and was estimated to contribute to 4.6 million deaths globally in 2017 alone [10]. Exposure to PM_2.5_ can increase the risk of numerous adverse health effects, such as lung cancer, preterm birth, and cardiovascular disease [11–13]. In a 2019 analysis of the Global Burden of Disease study, Yang et al. (2021) similarly documented a dramatic increase in the number of deaths between 1990 and 2019 due to chronic obstructive pulmonary disease (COPD) attributable to PM_2.5_ exposure [14].

Globally, the fastest growth in cases and age-standardized mortality rates for COPD and disability-adjusted life years attributable to PM_2.5_ has occurred in areas characterized by a low sociodemographic index [14]. Similar patterns were observed on a county-by-county basis as well, as illustrated by Verbeek et al. (2019), who conducted a spatial analysis in Ghent, Belgium, and showed an inverse relationship between income level and both noise and air pollution exposure (Verbeek, 2019), as well as Li et al. (2018), who reported a positive association between ambient PM_2.5_ concentrations and the social deprivation index in Hong Kong [15,16].

In the United States, a wide range of studies have similarly documented disproportionate exposure to air pollution and other environmental hazards among low-income communities and communities of color, both in California and nationally [17–25]. Regarding PM_2.5_, Tessum et al. (2021) showed a systemically disproportionate burden of exposure among people of color, which was evident across nearly all major emission categories and was consistent across states, urban and rural areas, income levels, and exposure levels [26]. Importantly, evidence has shown that the harmful effects of PM_2.5_ on life expectancy are exacerbated in states with higher income inequality and greater African American populations [27]. What is more, while overall air pollution levels have generally decreased in the U.S. over time, the disparities in PM_2.5_ and NO_2_ exposure have increased in some areas, underscoring gaps that still remain in regard to equitably reducing air pollution levels [28]. Importantly, since people of color are more likely to live in communities characterized by higher ambient PM_2.5_ concentrations, they are also more vulnerable to numerous health conditions, including asthma and COVID-19.

Over the course of the coronavirus pandemic numerous studies have found ambient air pollution to be positively associated with the prevalence and spread of COVID-19 as well as increased COVID-19 case fatality rates [29–34]. In one study it was estimated that over 14,000 lives lost due to COVID-19 as of July 2020 could have been avoided in the United States were it not for ambient air pollution originating from vehicle traffic and other sources [31]. What is more, socioeconomic disparities in infection rates, hospitalizations, and case fatality rates related to COVID-19 have been widely reported in the United States and globally. For instance, Moore et al. (2020) reported the COVID-19 case rate in the U.S. to be higher among African American and Latinx communities, while other studies have similarly shown African American, Latinx, and indigenous residents to be two- to four-times more likely to die from COVID-19 compared to Whites, with death rates in some states as high as 18-times that of Whites [35–39]. While there are a variety of socioeconomic and environmental factors, such as work and living environments, that limit social distancing and access to quality healthcare, contributing to these disparities, the disproportionate exposures and impacts related to air pollution cannot be overlooked.

In an effort to better characterize air pollution and other environmental hazards where government agency data are lacking, scientific researchers along with community leaders and residents increasingly collaborate in the development of community-engaged and participatory research methods for environmental and public health. Such research methods aim to involve community members, including minority groups, in every step of the research process, from developing research aims and collecting data to the dissemination of results [40,41]. Important goals of community-engaged research are to make scientific research more accessible, inclusive, and democratic, to share knowledge among researchers and impacted communities, and to position communities to leverage science to advocate for policy change. What is more, this collaborative approach acknowledges the fact that environmental hazards such as air pollution cannot as easily be addressed by research alone, requiring the mobilization of communities to influence public policy in their own areas. To date, community-based participatory research has engaged a diverse range of underserved groups, including indigenous, African American, and Latinx communities, as well as homeless youth and primary school students, and has focused on waste disposal practices, soil contamination, and indoor as well as ambient air quality, along with other exposure- and health-related concerns [41–44].

In terms of community-engaged research and air pollution, data collection methods have used both passive samplers (no electricity needed) as well as active portable air monitors. One study conducted in Barcelona, Spain involved participants from primary schools who measured NO_2_ concentrations using stationary passive samplers [45]. Another more recent study measured PM_2.5_ and radon in the homes of those living in the Rocky Mountain West tribal reservations [46]. One study that used portable air monitors was that by Johnston et al. (2019), who partnered with environmental justice organizations in Los Angles in order to outfit 18 youth participants with personal PM_2.5_ monitors so that they could characterize a typical “day in the life” in terms of air pollution across four neighborhoods [47]. In London, Varaden et al. (2021) involved 258 children across five primary schools by outfitting their backpacks with built-in air quality sensors capable of measuring their PM_2.5_ and NO_2_ exposures [48]. These latter studies both included educational workshops and community forums, and were found to be effective in teaching children/youth about their personal air pollution exposure and health implications.

In measuring air pollution, recent technological innovation has led to the development and deployment of thousands of low-cost air pollution sensors around the world, which have enabled scientists to characterize air quality at a high spatial and temporal resolution [49–52]. Such measurements represent an improvement upon traditional government-operated monitoring stations, which have historically suffered from uneven and sparse distribution that has limited their ability to measure air pollution variability at a local scale [53]. A recent study of air pollution data in California, for instance, showed low-cost sensors to offer an improved representation of PM_2.5_ spatially when compared to regulatory monitoring stations [54]. What is more, given their superior ability to identify air pollution hotspots, such sensors allow for more accurate air quality index reporting during wildfires and other extreme air pollution events, including those related to industrial emissions [52,55]. Given their affordable prices, mobility, and ease of maintenance, low-cost sensors can be owned and operated by governments, organizations, and individuals alike, which has helped this technology to expand regionally where government sensors have not, as well as enabled everyday citizens to actively participate in air pollution data collection and awareness.

In this pilot study we partnered with a non-profit founded by the Madison Park Neighborhood Association (MPNA), along with community volunteers, in order to measure local air quality throughout Santa Ana, California, using a validated low-cost air-monitoring device called the AtmoTube Pro. Our specific aims included: (1) characterizing air pollution near the industrial corridor in Santa Ana; (2) identifying potential air pollution hotspots and emissions sources using both mapping techniques and local community knowledge; and (3) characterizing and comparing air pollution within socially vulnerable areas versus those measured in less vulnerable communities within and outside of Santa Ana, so as to evaluate the potential of environmental inequities. We hypothesized that socioeconomically disadvantaged communities would contain the highest concentrations of air pollution.

The mobile air-monitoring approach used in this study, and its application to characterize the ambient air quality within a defined geographic region through predesignated walking routes and grid-like site assignments, is in contrast to other community-engaged studies, which tend to employ fixed-site monitoring and/or focus on personal air monitoring that is not bound geographically [56]. What is more, this study aims to offer insights about ambient air quality and potential source contributions that cannot be understood from fixed regional government monitoring stations. This is distinct from other community-engaged studies that focus on characterizing personal exposure [47,48].

## 2. Methods

This pilot study was conducted as part of a community–academic partnership involving the University of California, Irvine, and a local California-based community organization called MPNA. MPNA has been serving community members of Southeast Santa Ana, California, for over 30 years. In 2012 MPNA founded a nonprofit called GREEN-MPNA, the prefix of which stands for Getting Residents Engaged in Empowering Neighborhoods. GREEN-MPNA and its programs emerged from specific needs identified by Madison Park residents, including support for youth and families regarding access to educational and leadership opportunities as well the need to improve health outcomes through health education and the establishment of a safe and clean environment. Led by residents of Southeast Santa Ana, GREEN-MPNA in recent years has been studying environmental justice and related health risks associated with air pollution facing local residents.

GREEN-MPNA’s expansion into environmental justice issues began in 2017, when a group of residents received notices alerting them to the siting of a new metal plating facility across the railroad tracks from their apartments, which was also within 1000 feet of two elementary schools. With a grant from the California Air Resources Board, GREEN-MPNA convened the Comunidad Unida, Aire Limpio (CUAL) resident steering committee, made up of adult residents and high school youth, to learn about and investigate the environmental and public health risks near Madison Park. In 2018–2020 CUAL committee members participated in a two-year environmental justice training curriculum that included topics on community-based research, participatory mapping, developing an advocacy campaign, air pollution health risks, participating in public comment processes, and designing an air-monitoring project. During this period GREEN-MPNA and the CUAL committee identified the need for a community air-monitoring project to characterize the environmental and public health risks facing their community.

In 2019–2020 the CUAL committee used participatory processes, including workshops and focus groups, to identify members’ priorities for such a project, which included the need to identify major local pollution sources, compare environmental and health risks in Madison Park to other parts of the city, and understand personal health risks from exposure to air pollution. These specific research questions led to the current community–academic partnership that gave rise to the current pilot study, as well as the specific research questions that this this study was designed to answer.

The actual establishment of the community–academic partnership dates back to 2003, when MPNA began hosting community events that drew the attention, and ultimately the involvement, of UC Irvine faculty, which turned into ongoing collaborations involving clinical trials, educational outreach, etc. Thus, UC Irvine collaborators were a natural fit when it came to the current study. In terms of workflow, online meetings were first convened between academic and community partners in order to define community concerns and research questions as well as draft a study design that could be applied in the field so as to enable a formal data analysis. Upon presenting the finalized study plan to the wider community, residents were able to volunteer for field data collection and attend a subsequent training session. On each sampling day, trained volunteers received monitoring devices along with specific instructions regarding their assigned sampling locations. Following each monitoring day, all monitoring devices were transferred to academic partners for data exporting and compilation. All data analyses and statistics were carried out by expert research partners from UC Irvine.

### 2.1. Study Region

**Figure 1.**
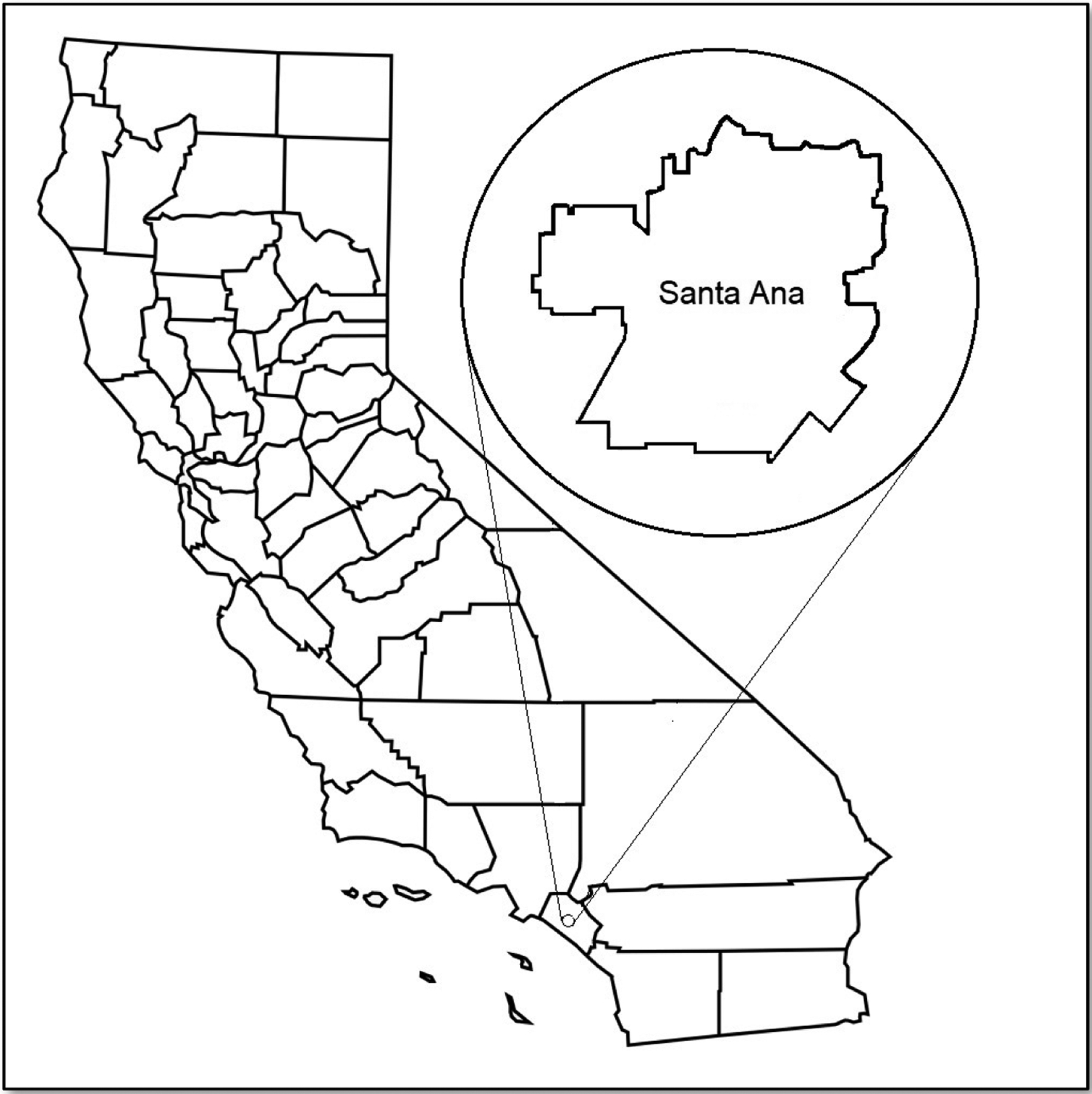
Map depicting the city of Santa Ana within California.

Santa Ana is a densely populated city located in Southern California in the southwestern region of the United States. It is the administrative center of Orange County, which is the sixth most populated county in the U.S. With a total population of approximately 337,716 residents, Santa Ana spans an area of 70.6 km^2^ and includes 61 census tracts [57]. In terms of population, Santa Ana ranks as the second largest city in Orange County and is the eleventh largest city in the state of California [57]. The majority of Santa Ana residents identify as Latina/o/x (77.3%), followed by Asian (11.4%) and white (9.4%), with a relatively high proportion (45.2%) of residents being immigrants [58]. As of 2019 the city includes 78,563 housing units and has a median household income of USD 65,313 (2018 dollars) [57]. A schematic showing the location of Santa Ana within the state of California is presented in Figure 1.

As a major urbanized area bordered by three major freeways, including the interstate 5 and 405 freeways, state routes 22 and 55, as well as the John Wayne Airport, potential sources of ambient air pollution in Santa Ana include both roadway traffic and aviation. Santa Ana is also an industrial and commercial center with over 26,432 businesses, including many metal-related industries (i.e., metal fabrication, metal cutting, and metal processing) [59]. Thus, point source emissions related to industrial activity and other facility operations represent additional potential contributors. Of particular concern to MPNA community members in regard to potential air pollution exposure is Santa Ana’s industrial corridor, which is an approximately 3 km^2^ neighboring area of dense industry that runs between South Standard Ave. and South Grand Ave., as well as between East 1st Street and East Warner Ave.

### 2.2. Field Sampling

From February to May 2021 a total of four air-monitoring field sampling days (one per month) were carried out across the general Santa Ana city area during three separate times of the day, which included morning (7–10 a.m.), midday (12–3 p.m.), and evening (4–7 p.m.). Sampling was conducted in the middle of the work week (Tuesday–Thursday) in order to capture peak traffic- and industry-related air pollution emissions and avoid potential holidays, which often occur on Mondays and Fridays. Air sampling was conducted by CUAL committee members and other trained community volunteers who were outfitted with AtmoTube Pro personal air pollution monitoring devices (AtmoTech, Inc., San Francisco, CA, USA) as well as Global Positioning System (GPS) devices called Qstarz^®^ Travel Recorders (QStar Technologies, Inc., Denver, CO, USA) in order to measure outdoor PM_2.5_ concentrations and their corresponding measurement times and locations. Prior to field use, all AtmoTube devices were recalibrated to ensure quality measurements. During field use the devices, which were attached to a carabiner and neck strap, were either worn around the neck, affixed to a belt loop, or held in hand so as to remain unobstructed and exposed to the ambient air.

In total, 82 community volunteers participated in this pilot study, 70 (85%) of whom were adults (18 years of older), with the remaining 12 (15%) being youth that were of high school age (14 to 17 years). Depending on the day, the number of field participants involved in any single monitoring day ranged from 30–40 volunteers, some of whom participated across multiple sampling days. While participants involved in data collection mostly consisted of members of the previously described CUAL committee, others consisted of community volunteers who learned about the project either through word of mouth or through social media outreach (e.g., digital flyers circulated through Facebook and Instagram). At the start of each field collection day a central check-in station was established at a local school campus, where community members met to receive and return their sampling devices in addition to field data collection sheets prior to and following, respectively, each sampling shift, as well as to receive specific instructions regarding their assigned sampling tasks.

For participants involved in data collection, field training included a live expert-led video tutorial (via Zoom) explaining how to properly use the air pollution and GPS measurement devices as well as how to properly use the written field data collection sheets (used as a backup in the case of GPS failure and to log active measurement periods). The video session was followed by a live question and answer period. Participants who could not attend the video session were trained in-person individually prior to data collection.

While capable of measuring volatile organic compounds (VOCs) and multiple size fractions of particulate matter (PM), the AtmoTube Pro (henceforth, “AtmoTube”) is best suited to measure concentrations of PM_1_ and PM_2.5_ as well as temperature and humidity [60,61]. Equipped with an optical PM sensor, the AtmoTube measures PM using a measurement principal that is based on laser light scattering [62]. Measurements are collected after first actively drawing air into the device using an internal fan [62]. The AtmoTube recently underwent field evaluation by the South Coast Air Quality Management District (SCAQMD) and demonstrated a high measurement accuracy for the detection of ambient PM_1_ and PM_2.5_ concentrations when compared to Federal Equivalent Method (FEM) instruments (R^2^ = 0.79–94) [60]. Given this government validation, and since PM_2.5_ is a regulatory air pollutant that has been linked with numerous adverse health outcomes, measurements of PM_2.5_ are the focus of this pilot study. For added quality assurance in this study, six pairs of AtmoTube devices (12 devices total) were collocated next to one another for at least six hours each throughout the study period, with their 10 min average PM_2.5_ measurements being compared by way of Pearson correlation coefficients.

#### 2.2.1. Air Pollution Hotspot and Source Detection

**Figure 2.**
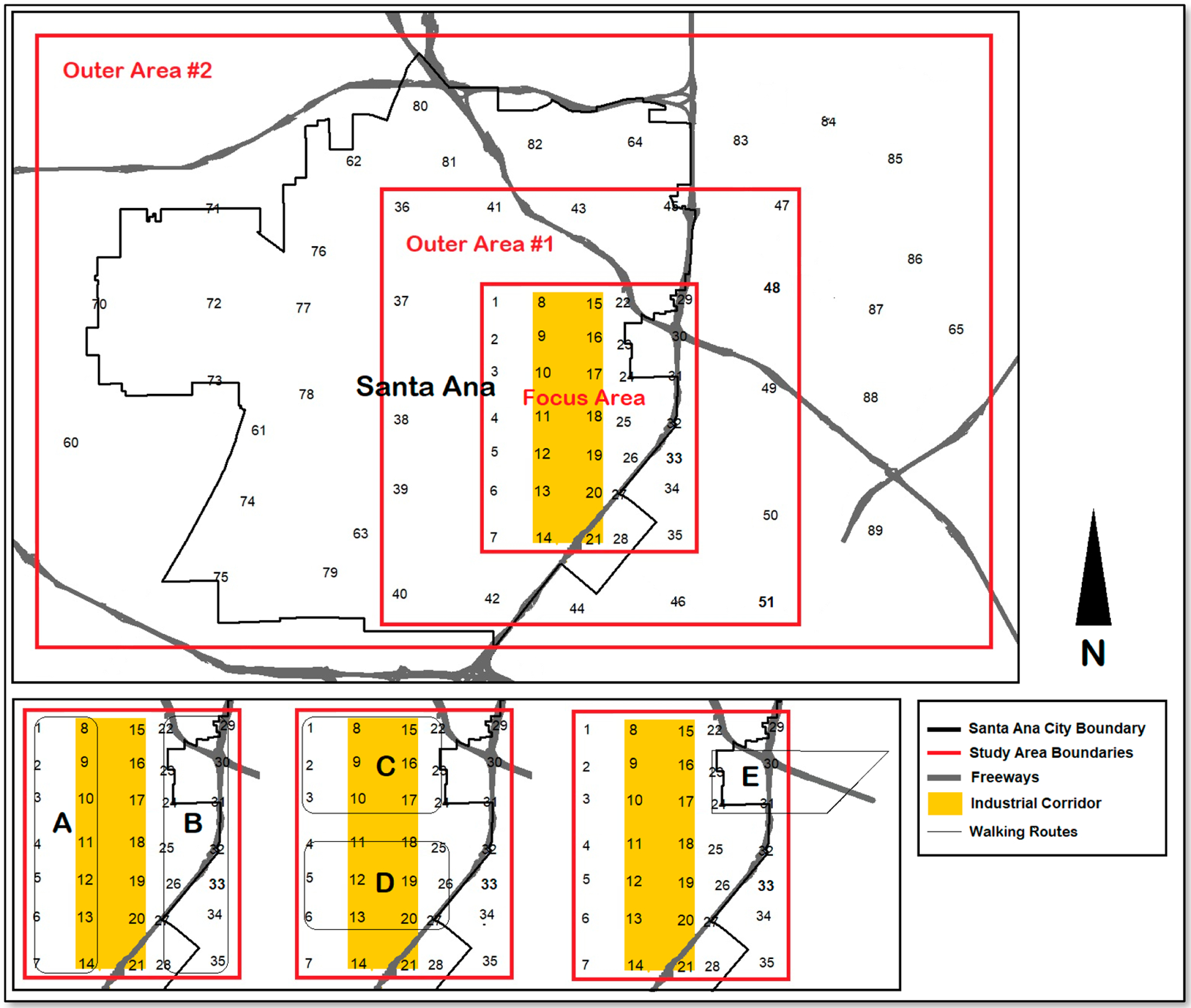
Map depicting regional sampling sites along with both the industrial corridor walking routes (**A**–**D**) and the freeway walking route (**E**).

Sampling sites were based on community input and feasibility. Since a primary concern expressed by community members was potential air pollution originating from the industrial corridor, four air monitoring routes (A–D) were established that encircled various sections of the industrial corridor along with one route (E) that encompassed the freeway system. Referred to collectively as the “walking routes”, these routes were designed for walking or biking since many community volunteers did not have access to an automobile. These routes were intended to enable the detection of both general air pollution around the industrial corridor as well as potential air pollution hotspots. To this end, community members carried handheld AtmoTube devices while walking along each of the five prescribed air-monitoring routes. These five routes are depicted in Figure 2. Each route was walked in its entirety once per sampling period, with three sampling periods (morning, midday, and evening) being carried out for each of the four monthly monitoring days (Feb–May). Beginning on the second of the four monthly sampling days, community members were outfitted with the GPS tracking devices that enabled a 15 s measurement of their exact GPS location within a 1 m resolution.

#### 2.2.2. Air Pollution Spatial Distribution

To characterize air pollution in a way that was less spatially biased than the walking routes, and therefore to enable a better regional comparison of air pollution between different regions within and outside of the city, we constructed a multitiered sampling grid across the study area that included the measurement of air pollution across 81 sites both inside and outside of the industrial corridor during all three sampling periods (morning, midday, and evening) and across all four monitoring days (February, March, April, and May). At the center of the map was a roughly 0.8 × 0.8 km sampling grid that spanned an area of 4 by 6 km and included 35 sampling sites. We called this the Focus Area and assigned it the highest sampling density since it encompassed the Madison Park community and industrial corridor, which is the area that was of primary interest to GREEN-MPNA as it relates to air pollution and exposure.

In a concentric square around the Focus Area was Outer Area #1, which consisted of a perimeter of 16 more sampling sites that were set 2 km beyond the Focus Area and spaced 2 km apart from one another. Finally, Outer Area #2 consisted of an additional 26 sampling sites spaced roughly 1.5–2.0 km apart from one another and which spanned the western and northern areas of Santa Ana (which were the most distant from the Focus Area), as well parts of the neighboring cities of Tustin and Irvine.

Each site was measured for 7–10 min by an AtmoTube device. Participants who collected air pollution measurements carried time-activity sheets through which they recorded the exact times of their sampling at each assigned site. Participants were also asked to abstain from any air-polluting activities, such as cigarette smoking, while sampling and to record the timing of such activities if/when they occurred so as to allow for the proper interpretation of data (no smoking was reported). Regional sampling was carried out using a combination of walking, biking, and driving. Where observations were collected by car, participants were asked to turn off their car engine and exit their vehicle during the sampling period in order to prevent measurements from being influenced by their own vehicle emissions.

To serve as baseline air pollution measurements, we identified sites that were more distant from the urban environment and therefore less influenced by anthropogenic air pollution sources (e.g., traffic, industry, etc.). Specifically, we identified four sampling sites outside of Santa Ana, which included sites in Irvine Regional Park, Santiago Canyon, Newport Beach, and the Newport Bay, located within the same county (Orange County, CA). Figure 2 presents depictions of the walking routes and regional sampling sites, as well as identifies the locations of both the industrial corridor and nearby freeway system. Due to the scale of the map, the four baseline sites could not be included in Figure 2 and are instead depicted in Figure S1 of the Supplemental Materials section.

When examining the spatial distribution of air pollution within Santa Ana, we compared measurements collected within (or contiguous to) and outside of the industrial corridor as well as measurements collected immediately next to (<100 m) and not next to the freeways system. Additionally, we compared measurements averaged across sites located within (or contiguous to) so-called “environmental justice communities,” or EJ communities, and those collected within non-EJ communities. The city of Santa Ana defines an EJ community as an area of the city where residents have the highest risk of exposure to air, water, and soil pollution and are burdened by socioeconomic and health issues, such as higher rates of language barriers, poverty, and asthma [63]. The most recently updated EJ community map is provided as Figure S2 in the Supplemental Materials section.

### 2.3. Statistical and Spatial Analysis

In order to carry out statistical and spatial analyses for this pilot study, .csv files were first exported from both the AtmoTube devices and Qstarz GPS devices. In the case of the AtmoTube, air pollution measurements were recorded as 1 min averages accompanied by a 1 min time stamp. To determine which datapoints to retain for analysis each AtmoTube’s datasheet was reviewed alongside its corresponding handwritten field data collection log (indicating exact time ranges when devices were collecting data at each assigned location). All rows (1 min time stamps) that did not correspond to a time of “active monitoring,” as indicated by the times recorded in the field data logs, were assumed to be periods of inactive monitoring (e.g., participants traveling between assigned monitoring sites) and were therefore discarded. Of note, the discarding of such between-site measurements was only necessary for the outer sites where participants required a car to travel from site to site (measurements by car were not desired given the potential of the participants’ own car exhaust to influence the measurements).

During data cleaning, all rows of data were also assigned a unique code indicating which, if any, of the A-through-E walking routes they collected data along. In regard to such routes, where participants collected data by foot or bike and carried a GPS monitoring device, an important step for analysis was the matching of AtmoTube air-monitoring measurements with GPS location measurements. In contrast to the 1 min average AtmoTube measurements, however, the Qstarz GPS device recorded GPS measurements every 15 s. In order to match the measurements of a given AtmoTube with its corresponding GPS device’s measurements it was therefore necessary to first convert the 15 s GPS data into 1 min averages (i.e., averaging the latitude and longitude coordinates to yield an average location). Once the 1 min air pollution and GPS measurements were matched the data were then imported into ArcGIS software where the locations of each air pollution measurement could be projected spatially onto a map for visual representation. In regard to summary statistics, all calculations and analyses were performed using SAS software [64].

## 3. Results

The following presents results from 10,972 one-minute average PM_2.5_ measurements (~183 h) collected across four monthly sampling days, each of which included a morning, midday, and evening sampling period in the city of Santa Ana, CA. The average PM_2.5_ concentration measured across all sampling days and all measurements collected regionally and along walking routes (A–E) was 6.9 μg/m^3^, with a standard deviation of 6.1 μg/m^3^ and a maximum of 90 μg/m^3^. Average concentrations categorized by month are presented in Table S1 of the Supplemental Materials section.

### 3.1. Air Pollution Hotspot and Source Detection

**Figure 3.**
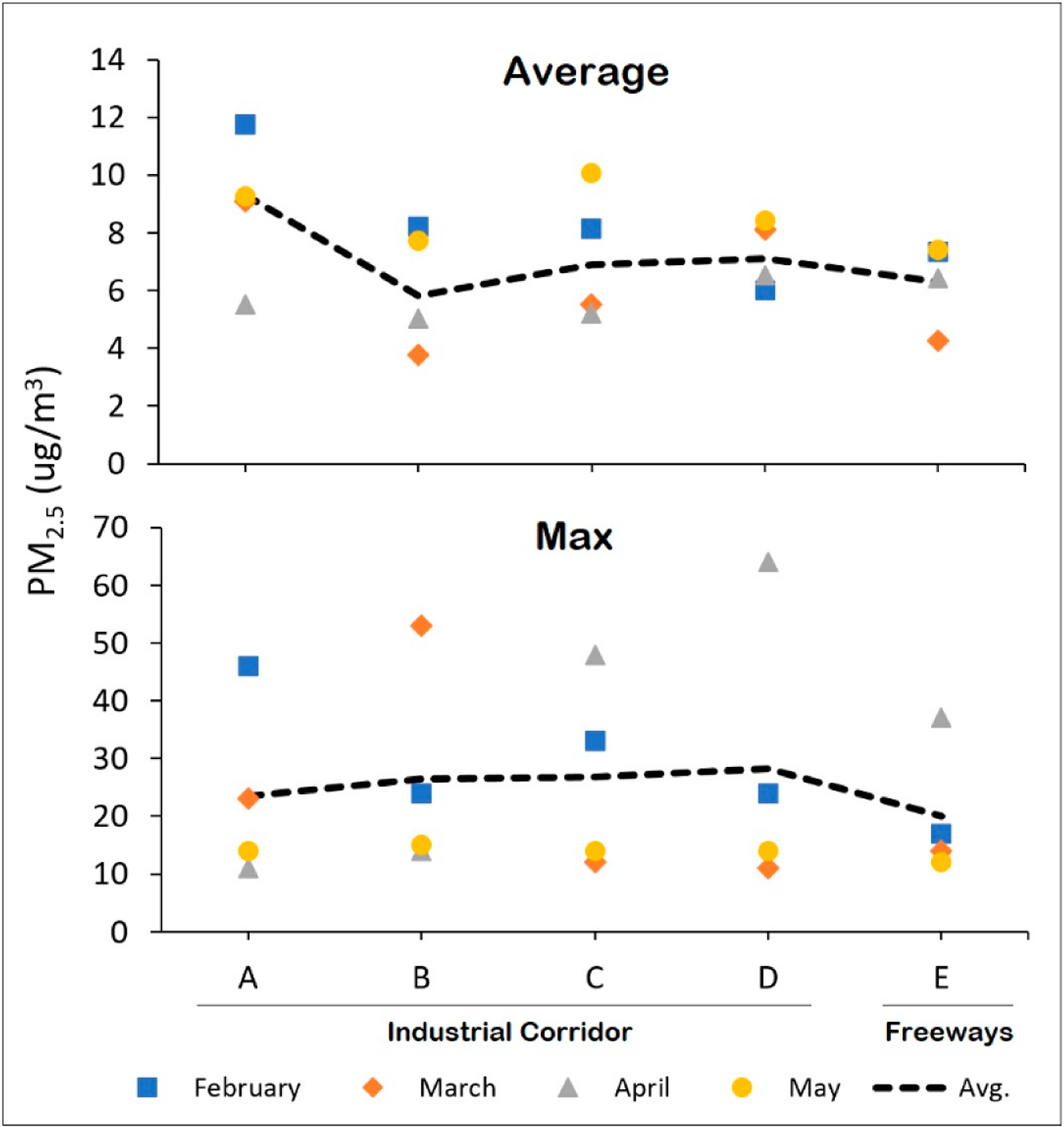
Average (over entire sampling day) and one-minute maximum PM_2.5_ concentrations across each of four monthly sampling days and across each of five walking routes (A–E) measured simultaneously.

Figure 3 presents the average and maximum PM_2.5_ concentrations across each of the four monthly sampling days and across each of the five walking routes. As shown in Figure 3 and summarized in Figure S3, the industrial corridor routes A and D were most frequently ranked the highest in terms of average PM_2.5_ concentrations when compared to all other routes within a given sampling day, respectively, while industrial corridor route B was most frequently ranked the highest in terms of maximum PM_2.5_ pollution (not shown in the graph). In terms of absolute concentrations, route A showed the highest average PM_2.5_ level (11.7 μg/m^3^), which took place during the February sampling day. Of note, this value is slightly below the U.S. Environmental Protection Agency’s (EPA’s) primary annual fine particle standard of 12.0 μg/m^3^. Route D showed the highest one-minute maximum PM_2.5_ level (64.0 μg/m^3^), which took place during the March sampling day. Route E, which encompasses the freeway system, was ranked lowest (along with route B) in terms of relative PM_2.5_ concentrations.

**Figure 4.**
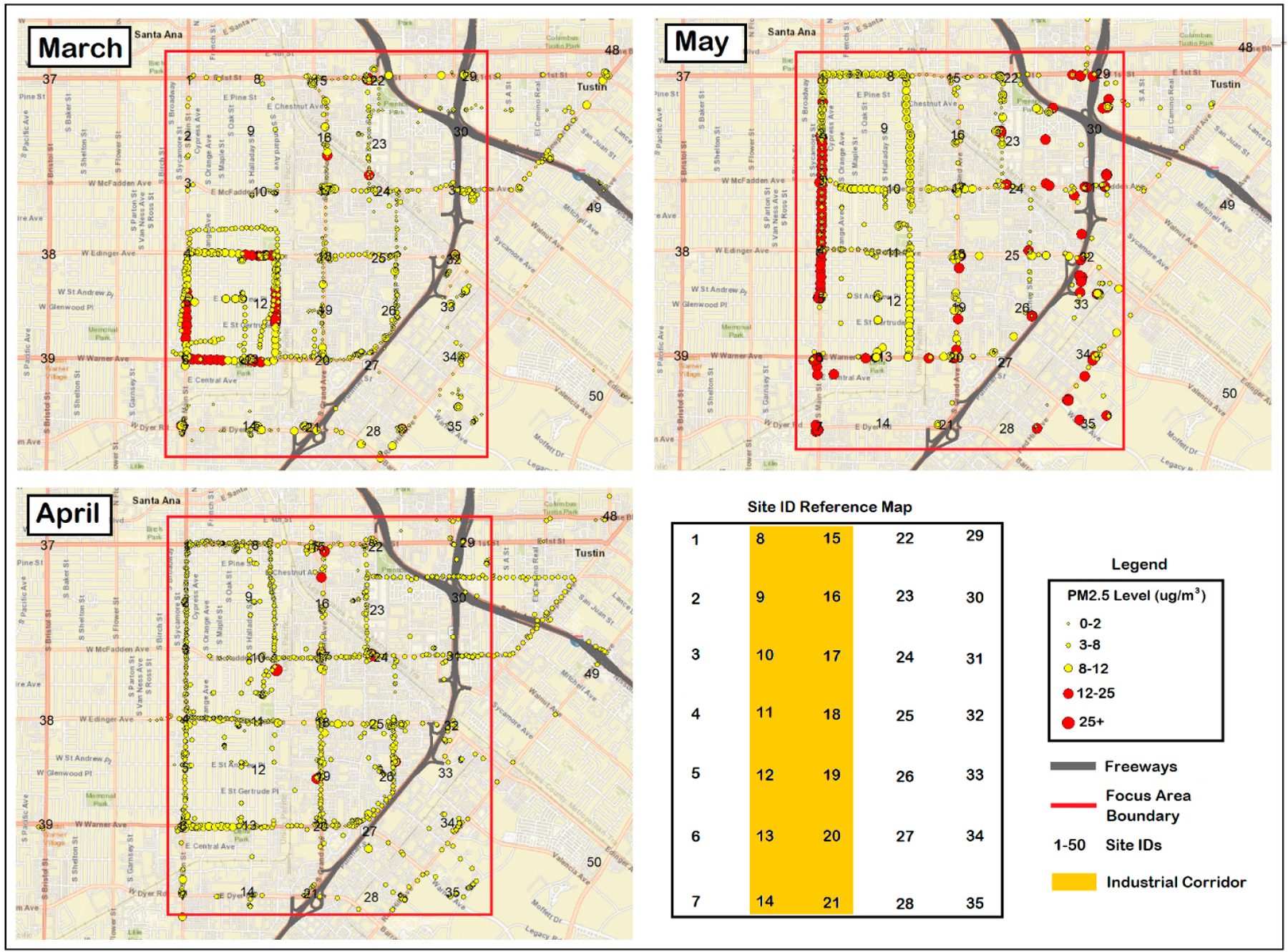
One-minute average PM_2.5_ measurements projected across the Focus Area in Santa Ana using high-resolution GPS tracking devices. Red dots denote PM_2.5_ > 12 μg/m^3^.

Figure 4 presents one-minute average PM_2.5_ measurements projected across the Focus Area in Santa Ana, demonstrating the utility of using high-resolution GPS tracking devices for field air monitoring and the detection of potential air pollution hotspots and/or heavy emission sources. In general, the majority of elevated PM_2.5_ concentration measurements (>12 μg/m^3^) occurred in March as well as May and corresponded with the industrial corridor area, as shown by the extensive red dots along Main Street and Standard Avenue in the western portion of the maps. Scattered exceedances also occurred near the freeways in May, whereas elevated freeway levels were not visible in March. In April, concentrations only exceeded 12 μg/m^3^ at a few scattered locations along the middle and northern industrial corridor. Otherwise, the distribution of air pollution in April tended to show higher levels near the southwestern quadrant of the map, near sites 6 and 13, corresponding to the southern end of the industrial corridor. GPS data for February are not depicted since such data were not available for this month. A presentation of each map broken down by morning, midday, and evening measurements for each monthly sampling day is presented in Figure S4 of the Supplemental Materials section and demonstrated relatively higher PM_2.5_ concentrations near roadway intersections.

### 3.2. Air Pollution Spatial Distribution

**Figure 5.**
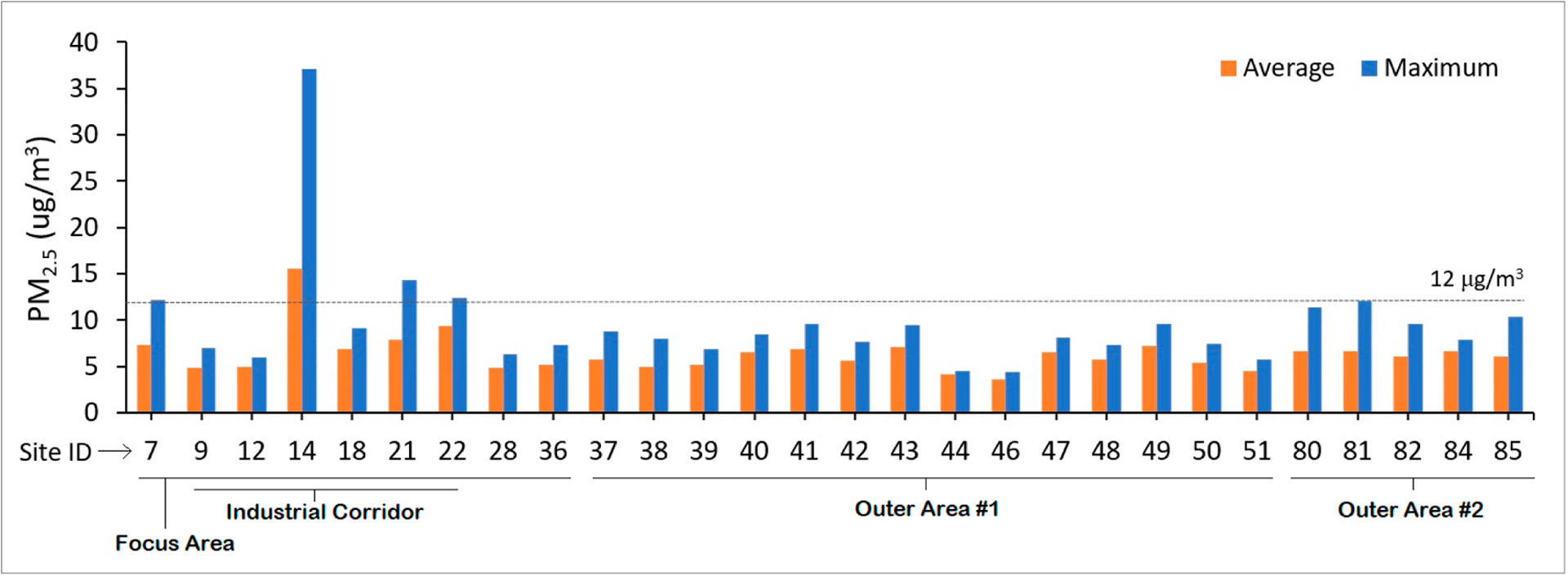
February sampling day, where average and maximum PM_2.5_ concentrations were found to exceed 12 μg/m^3^ across four sites.

Figure 5 illustrates the February sampling day, where the average and/or maximum PM_2.5_ concentrations were found to exceed 12 μg/m^3^ across four sites within the Focus Area compared to zero sites outside of the Focus Area. This graph only includes sites that were successfully measured during all three sampling times within a day so as to avoid time-of-day sampling bias. As shown, site 14 appears to be anomalously high relative to other sites. This site was located within the Focus Area of the study region, which is immediately adjacent to the industrial corridor. Although Figure 5 only presents the February sampling day as an example, the May sampling day also showed one site (site 6) where the average and/or maximum PM_2.5_ concentration was elevated. Site 6 is also adjacent to the industrial corridor and was the only site where average and/or maximum PM_2.5_ concentrations were elevated across two separate monthly sampling days.

In general, 14 of the 24 (58%) high-pollution sites (PM_2.5_ > 12 μg/m^3^) fell within the Focus Area, while 17 (71%) fell within the Outer Area #1 boundary. More noteworthy is that 10 of these 24 sites (42%) fell along the industrial corridor, despite this area only accounting for 12% of the total sites sampled. In total, 13 of the 24 (54%) elevated sites showed average PM_2.5_ concentrations ≥ 12 ug/m^3^, which is the U.S. EPA’s primary annual fine particle standard.

**Figure 6.**
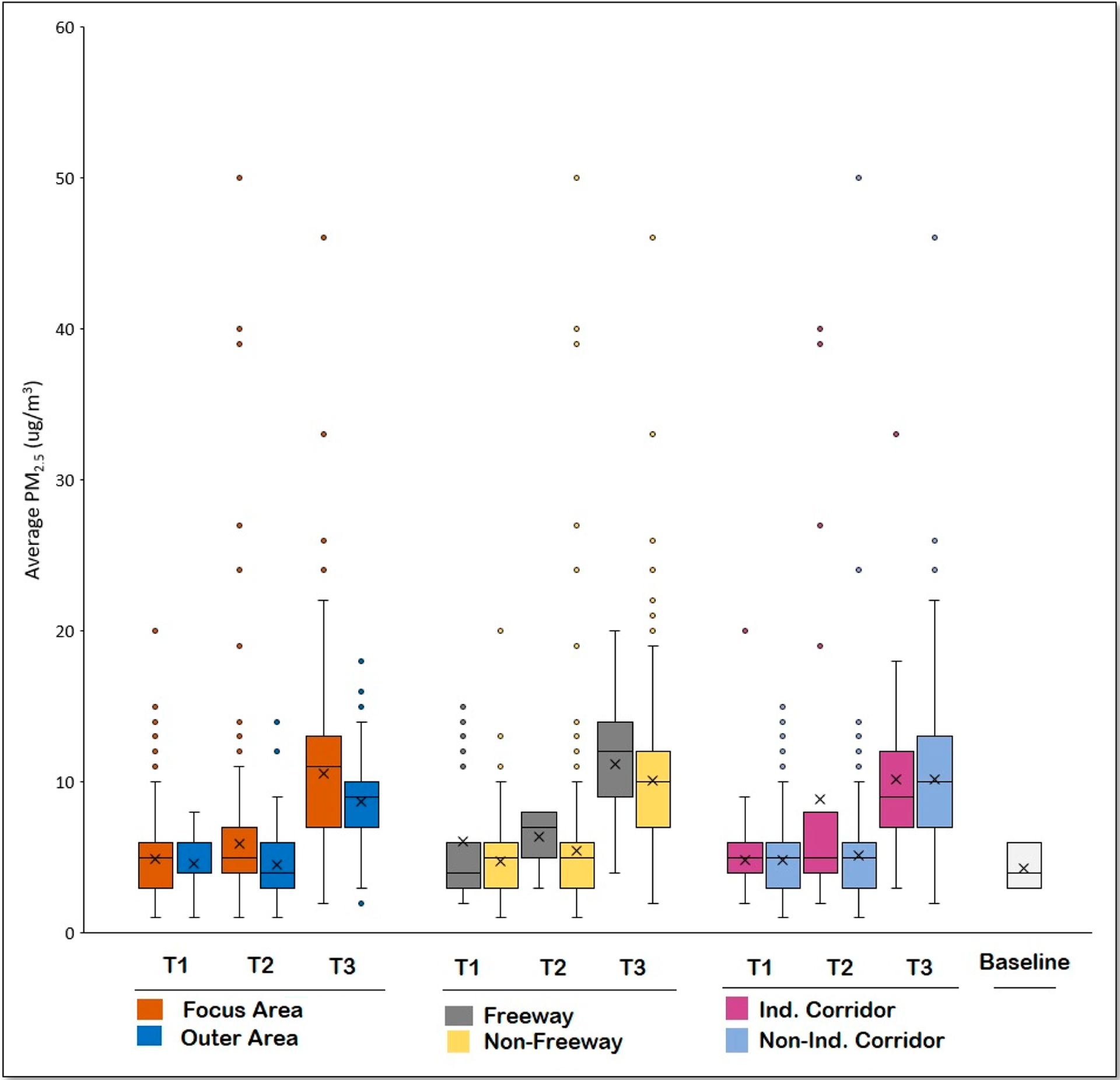
Boxplots of PM_2.5_ concentrations averaged across each time period (T1 = morning, T2 = midday, and T3 = evening) and relevant urban features for measurements collected on the February sampling day. The symbol “X” indicates average concentrations.

Figure 6 depicts boxplots of PM_2.5_ concentrations averaged across each time period and land area designation for measurements collected during the February sampling day. As shown, average concentrations were higher in the Focus Area, which encompasses the industrial corridor and MPNA region, relative to samples collected in the Outer Area (includes both Outer Area #1 and #2). The average PM_2.5_ concentration of samples collected near freeways was higher than those collected in nonfreeway zones during all three sampling periods, although nonfreeway areas exhibited more high-concentration outliers. When examining samples collected near the industrial corridor, the average concentration was substantially higher than the nonindustrial corridor average during the midday sampling period, but approximately equal during other sampling periods.

Although February was presented as an example, the pattern of PM_2.5_ between the Focus Area and Outer Area was similar in March and May, with an opposite pattern exhibited during the midday in April. In April, the pattern related to highway areas was also opposite, with nonfreeway areas showing higher average PM_2.5_ concentrations. For the industrial corridor comparison, results for other months conflicted with those of February, with non-industrial corridor areas showing higher concentrations during the midday for the March and April sampling days. In general, measurements collected in greenspace areas (not shown graphically) were variable, in some cases higher than those of Outer Area samples while in other cases lower.

**Figure 7.**
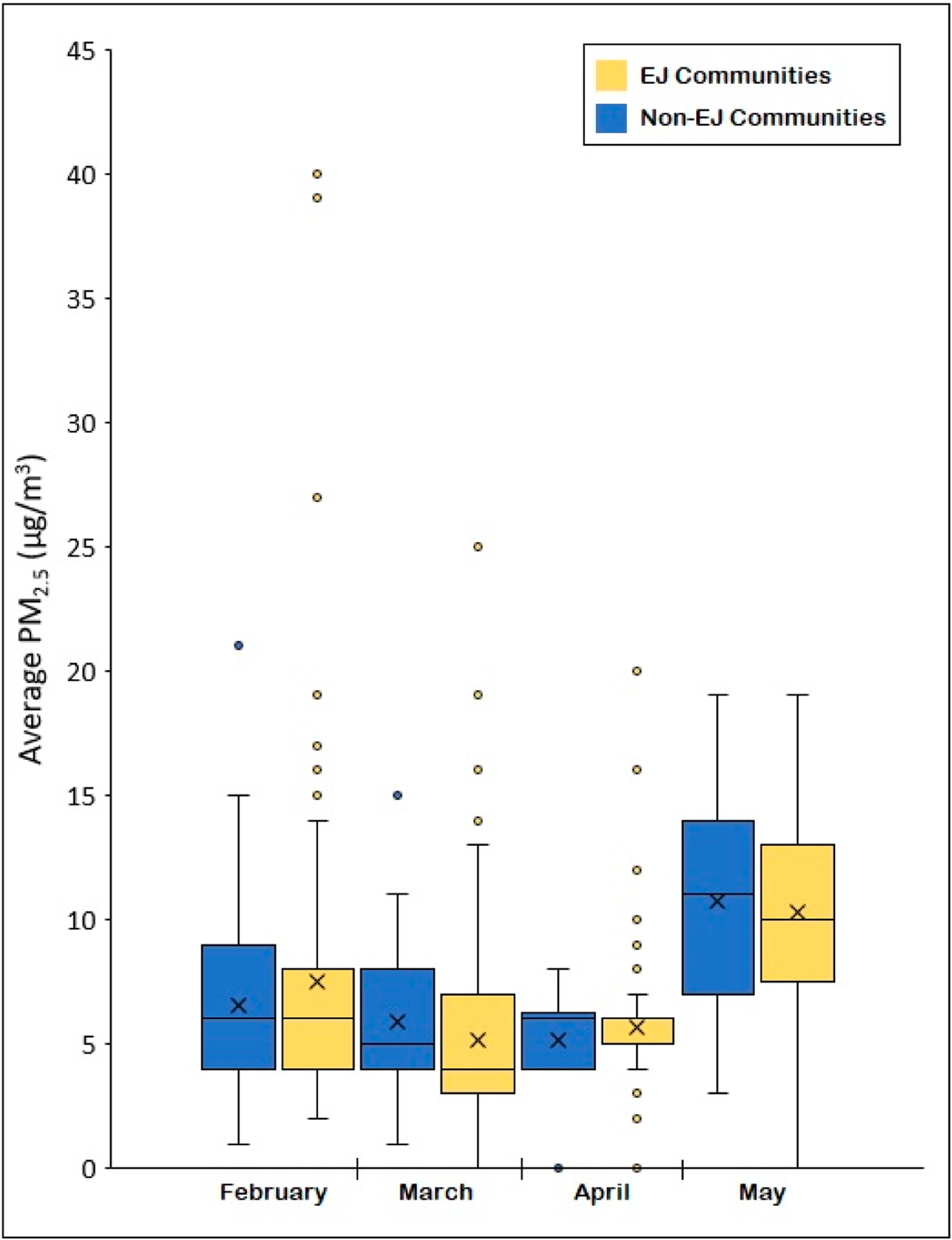
Boxplots of PM_2.5_ concentrations averaged across environmental justice (EJ) communities and non-EJ communities in Santa Ana for measurements collected across all four of the monthly sampling days. The symbol “X” indicates average concentrations.

Figure 7 presents a boxplot of PM_2.5_ concentrations averaged across measurements collected within EJ communities and non-EJ communities for each of the four monthly sampling days. As shown, average concentrations were not considerably different between EJ and non-EJ communities, with each community designation reporting a relatively higher PM_2.5_ average for two of the four sampling days. For EJ communities, however, the boxplots consistently demonstrate a greater frequency of high-PM_2.5_ outliers relative to non-EJ communities.

**Figure 8.**
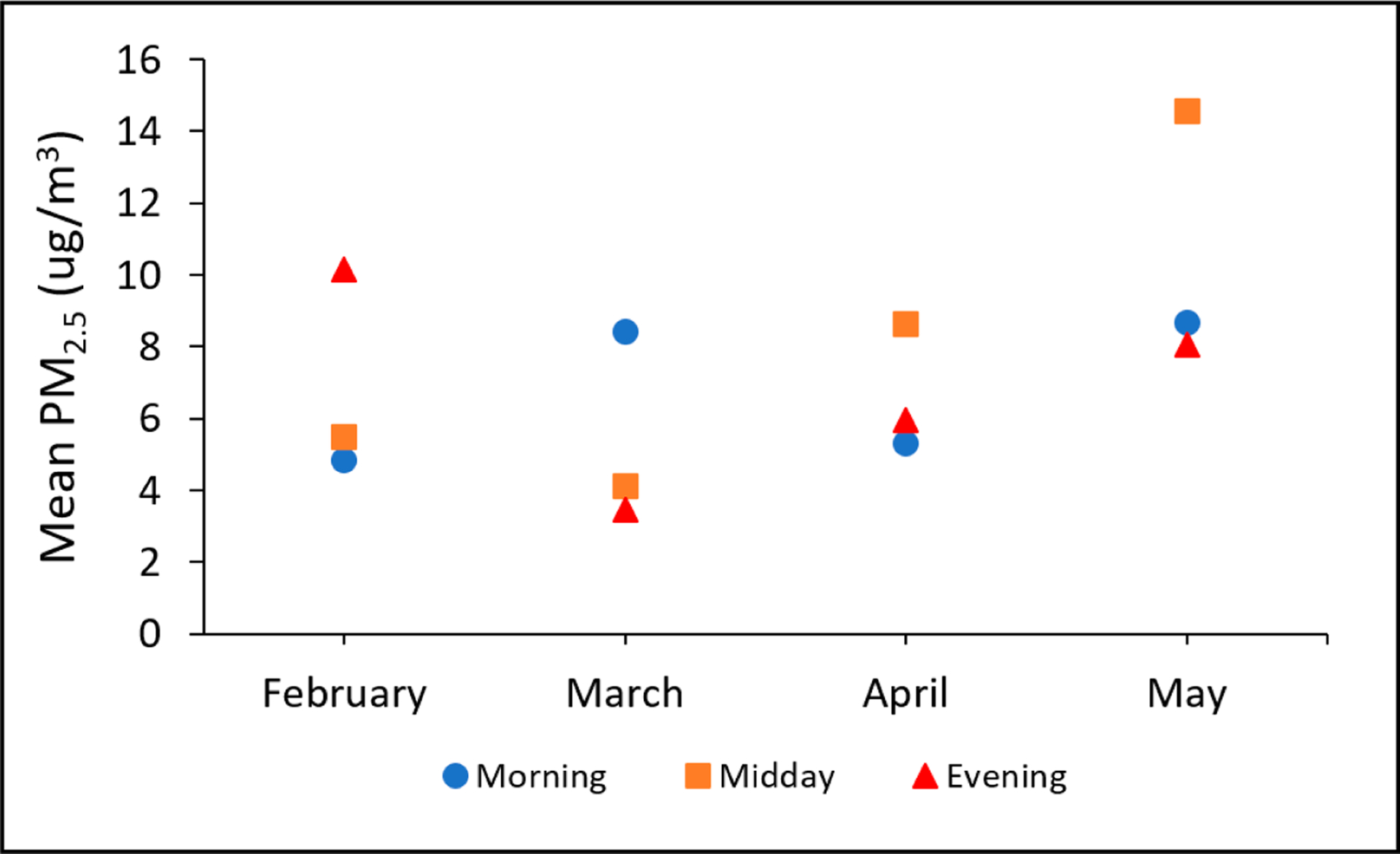
Average PM_2.5_ concentrations across each monthly sampling day and each sampling time period.

Figure 8 presents the average PM_2.5_ concentrations across each monthly sampling day and each sampling time period. As can be seen, average PM_2.5_ concentrations appeared to depend heavily on both sampling time and sampling day. Intraday variability ranged from approximately 4 to 9 μg/m^3^, which represented over a doubling in average PM_2.5_ in some cases. Similarly, intraday variability ranged from approximately 4 to 10 μg/m^3^. In the present study, the May sampling day had systematically higher PM_2.5_ concentrations.

In this study, limited colocation analyses performed across six paired AtmoTube devices demonstrated high Pearson correlation coefficients for five of the pairs (r = 0.71–0.91) when comparing 10 min averaged PM_2.5_ measurements, whereas one colocated pair showed only a moderate correlation (r = 0.42). Of note, the AtmoTube device also automatically measures and reports 1 min average PM_10_ and PM_1_ concentrations. However, the analysis of this data is not presented in this study since the concentrations of these two pollutants were very highly correlated with 1 min averaged PM_2.5_ concentrations (r = 0.97 and 0.91, respectively) and therefore did not add value to our understanding of the spatial distribution of air pollution.

## 4. Discussion

This pilot study employed a community-based participatory approach in order to understand PM_2.5_ air pollution levels in the city of Santa Ana, CA, and in turn address concerns of residents about the potential for unhealthy and disproportionately distributed exposures related to industry, traffic, and other sources. The results demonstrated PM_2.5_ concentrations to vary by region, with levels within the Focus Area being generally higher than measurements collected within more affluent areas in Santa Ana and the neighboring city of Tustin. The Focus Area is also the region characterized by some of the highest poverty rates and proportion of minority residents in Santa Ana, as well as the area that is nearest the industrial corridor. In general, the majority of elevated PM_2.5_ sites fell within the Focus Area, with nearly half falling along the industrial corridor despite this area only accounting for a minority of the total sites sampled. These findings suggest greater air pollution within this region of the study area and the likely contribution of industrial sources to local air pollution.

In developing a data collection protocol for community residents, walking routes were designed in order to enable community participants to collect PM_2.5_ measurements around the industrial corridor as part of an air pollution hotspot detection approach, while a grid-like design was pursued to enable a regional comparison of air pollution across the wider study region. Results from hotspot detection showed instances of exceptionally high PM_2.5_, while levels on average were relatively low, falling below the EPA’s annual ambient PM_2.5_ standard of 12 μg/m^3^.

When considering individual walking routes, routes A and D (encompassing the industrial corridor) were most frequently ranked the highest in terms of average PM_2.5_ concentration while route E (encompassing the freeways) was ranked relatively low when compared within a given sampling day. This suggests that the industrial corridor is likely to be a more important source of PM_2.5_ than freeway traffic.

These findings were affirmed when pairing one-minute average PM_2.5_ measurements collected along the walking routes with high-resolution GPS tracking data. Such results showed that the majority of elevated PM_2.5_ concentration measurements (>12 μg/m^3^) occurred along the industrial corridor, particularly along Main Street and Standard Avenue. The minor relative contribution of air pollution by the freeways was most evident when examining the March and April air pollution maps, in which low air pollution appears near the freeways compared to the higher levels along the industrial corridor. Nonetheless, PM_2.5_ concentrations of samples collected near freeways was on average higher than those collected in non-freeway zones (outside the industrial corridor area), thus affirming that freeway proximity still influences air pollution concentrations. Of note, the fact that this study took place during the COVID-19 pandemic may have resulted in lower-than-normal traffic and thus traffic-related emissions.

High concentrations measured via the walking routes corresponded to some of the same site IDs (e.g., sites 6 and 14) in which high air pollution was measured during regional sampling (as discussed below). Although not shown graphically, scrutiny of each map broken down by morning, midday, and evening measurements demonstrated modestly higher concentrations near roadway intersections, which is reasonable given the vehicle braking and acceleration that can produce particles [65].

When examining specific sites across which a full day of PM_2.5_ data was collected, sites 6 and 14 were found to be anomalously high relative to other sites. Although this study is not able to attribute air pollution to specific sources, it is worth noting that both of these sites fall within the industrial corridor boundary. What is more, a recent report using the same measurement devices (AtmoTube Pro) documented indoor PM_2.5_ concentrations within an industrial facility near these sites to be over 200 μg/m^3^, with average outdoor levels of up to 39 μg/m^3^ (avg. = 17 μg/m^3^) over a three-day period, thus underscoring the potential for nearby industrial sources to pollute the local ambient environment [66].

Boxplots of PM_2.5_ concentrations averaged across different time periods and relevant urban features showed concentrations to generally be higher in the Focus Area of the study, which encompassed the industrial corridor and MPNA community region, compared to surrounding areas. Average measurements collected in greenspace areas were mixed, with PM_2.5_ concentrations in some cases being higher than those of other land area types. Higher air pollution in greenspace areas could conceivably be a result of smoke-generating recreational activities such as barbequing, or could be due to unrelated neighboring air pollution emissions from industry or traffic. Of note, due to physical properties which lead to its slow removal time, PM_2.5_ has a relatively homogeneous spatial distribution compared to other air pollutants (e.g., NO_x_, CO, and ultrafine particles). This could result in greenspace areas downwind of major sources having high PM due to regional contributions.

When comparing PM_2.5_ measurements collected within EJ communities and non-EJ communities, average concentrations were not considerably different. However, EJ communities consistently demonstrated a greater frequency of high-PM_2.5_ outliers relative to non-EJ communities over the four sampling days. This suggests the increased presence of local air pollution sources and the need to better identify the causes of such emissions among EJ communities, particularly given the disproportionate socioeconomic disadvantages that such communities already face.

In regard to measurements collected near the industrial corridor, average concentrations were higher than the non-industrial corridor average in some cases, while the opposite pattern (or no significant difference) was observed in other cases. This may be explained in part by differences in industrial activity that occur during different times of the day (e.g., industry emissions may be low in the evening as industrial facilities begin to close for the day) as well as differences in meteorology. For instance, mild rainy weather the night before the first two sampling days may have depressed PM_2.5_ concentrations, as perhaps did moderate wind, which picked up midway through the second sampling day. What is more, stable conditions and sunny/hot weather encountered during the May sampling day may have contributed to the elevated measurements, perhaps due to photo-related secondary PM_2.5_ formation.

Of note, atmospheric chemistry, meteorology, and long-range transport are all important factors that affect local air quality. Importantly, however, given the small geographic domain of our study area, we anticipate these factors to result in a systematic influence on air pollution as measured across all monitors within a given time period, having only a minimal impact on the relative distribution of air pollution from one site to the next (which is the primary focus of this study and of community concerns). Having said that, the influence of regional factors is something that we considered in this study and attempted to account for by collecting baseline measurements that are minimally affected by local emissions sources (e.g., nearby industry, nearby vehicle exhausts, etc.).

Although there were insufficient data collected to enable an appropriate comparison with the EPA’s ambient air quality standards, it is nonetheless worth noting that the overall average PM_2.5_ concentration calculated across all four sampling days was not found to exceed 12 μg/m^3^, which is the U.S. EPA’s annual primary annual fine particle standard. However, the fact that multiple sites showed average PM_2.5_ concentrations that were above the EPA standard suggests the need for follow-up sampling in Sana Ana to determine if the site measurements in this study are reflective of annual average concentrations and therefore underscore the existence of a public health concern.

An important strength of this pilot study is its inclusion of community priorities in addition to its foundation built around community–academic partnerships [67–70]. The research questions, study design, study implementation, and continued envisioning of a healthier community were each guided by our partnership process. Community–academic partnerships characterized by ownership of action research agendas by community and academic partners better position communities to mobilize around action items so as to promote community health and health equity [67,70]. Another strength of this study is the grid-like sampling and near-simultaneous collection of measurements across a large number of sampling locations, thus allowing for a more spatially resolved understanding of the distribution of air pollution. This helps to reduce exposure misclassification. High-density spatial sampling also enabled an assessment of average air pollution concentrations across multiple land areas (e.g., freeway vs. greenspace). An additional strength is the characterization of air pollution across both industrial and nonindustrial areas, which was an important community priority as it relates to the identification of potential emissions sources and pollution hotspots.

This study had several limitations. First, despite a high number of sampling sites, a limitation of this study nonetheless was the inherent inability to characterize air pollution concentrations between sampling sites despite the variability that likely exists between such sites. Second, this study was limited to daytime measurements across four single monitoring days and therefore can neither characterize air pollution during nighttime hours nor during the full span of a given month or across different seasons. What is more, despite the simultaneous design of data collection during discrete sampling windows (e.g., morning), samples within a given sampling window were not collected exactly simultaneously, with samples within the same period potentially being collected up to three hours apart from one another (e.g., 7 a.m. and 10 a.m. are each considered morning samples). This may have introduced variability in PM_2.5_ concentrations that was due to temporal as opposed to spatial differences. Furthermore, while one of the aims of this study was to distinguish potential sources of ambient air pollution, the absence of wind trajectory data and speciation-based source apportionment data means that our conclusions cannot be considered definitive, but rather should be considered a first step at understanding the relative contributions of these potential sources.

Limitations related to the low-cost sensors and community-based participatory methodology of this pilot study should also be discussed. This includes the difficulty of sustained participation by volunteers, in turn resulting in different numbers of people being available for field data collection for each monitoring data. This resulted in variable amounts of data and undersampling during certain monitoring days and time periods, which required us to restrict our analysis to only “full day” observations that amounted to fewer than anticipated sampling sites. Given that many volunteers were non-English-speaking Latinx residents and also low income, an additional challenge was verbal communication between certain residents and university researchers, as well as the inaccessibility of certain residents to automobiles for field sampling. These challenges were able to be overcome, however, thanks to community leaders and researchers who translated between languages, as well as by tailoring the study design to include walking routes.

Another limitation is that the mobile nature of citizen-based measurements (as opposed to fixed monitoring stations) meant that high-air-pollution zones (i.e., hotspots) could not be entirely distinguished from mobile sources (e.g., a heavy-duty truck passing by). We attempted to overcome this through repeated measurements during the same day and over multiple days; however, this potential influence cannot entirely be ruled out given the limited data collected in this study. Additionally, while volunteers were trained in the use of measurement devices, at least three devices appeared to have been unknowingly shut off in the middle of field monitoring, which resulted in lost data. Lastly, in regard to data quality and the use of low-cost sensors, colocation analyses showed one of six paired devices to perform only modestly when comparing colocated measurements, suggesting that device reliability may present an issue for studies involving such sensors, despite calibration, and that colocation experimentation prior to field deployment may be warranted to identify faulty devices.

## 5. Conclusions

This pilot study employed a community-based participatory approach in order to develop community-derived research questions regarding air pollution levels in the city of Santa Ana, CA, and in turn address concerns of residents about the potential for unhealthy and disproportionately distributed PM_2.5_ exposures related to industry, traffic, and other sources. The results demonstrated PM_2.5_ concentrations to vary by region, with levels within the Focus Area being generally higher than measurements collected within more affluent outside areas. Similarly, measurements collected within EJ communities exhibited more high-PM_2.5_ outliers compared to non-EJ communities. When examining regional measurements, samples collected near freeway and industrial corridor sites tended to generally be higher than sites further from the freeways and industrial corridor, although this pattern was not consistent across all months and sampling periods. While PM_2.5_ concentrations exhibited regional variability, concentrations were on average below the EPA’s annual PM_2.5_ standard. Maximum concentrations recorded near the industrial corridor and within EJ communities nonetheless underscore the potential for air pollution concerns in certain regions, and therefore the need for follow-up monitoring. This pilot study demonstrates the utility of using low-cost air pollution sensors for the application of community-engaged study designs that leverage community knowledge, enable high-density air monitoring, and facilitate greater health-related awareness, education, and empowerment among communities. What is more, results from this study underscore the existence of environmental health inequities that persist in urban areas today. These findings help inform environmental-justice-related initiatives focused on community outreach, future urban planning, access to resources, and the formulation of community-driven recommendations for policy makers.

## Data Availability Statement:

Data available upon request.

## Supplementary Material

Supplementary Material
