# Supplementary material for: Community-Engaged Use of Low-Cost Sensors to Assess the Spatial Distribution of PM2.5 Concentrations across Disadvantaged Communities: Results from a Pilot Study in Santa Ana, CA
Source: Atmosphere (Basel). Author manuscript; Available in PMC 2022 Sep 30. (PMC9523797; doi:10.3390/atmos13020304)
Supplement: Supplementary Material [file NIHMS1797288-supplement-Supplementary_Material.pdf]

Supplemental Materials

Table S1. Summary PM2.5 statistics across all 4 monthly sampling days.

Averaging Period	N	Mean	Std Dev	Min	Max
All 4 Days	10,972	6.9	6.1	0.0	90.0
February	3,104	6.9	4.4	1.0	50.0
March	2,439	5.3	3.3	0.0	53.0
April	3,313	6.8	9.2	0.0	90.0
May	2,116	9.0	3.5	0.0	19.0

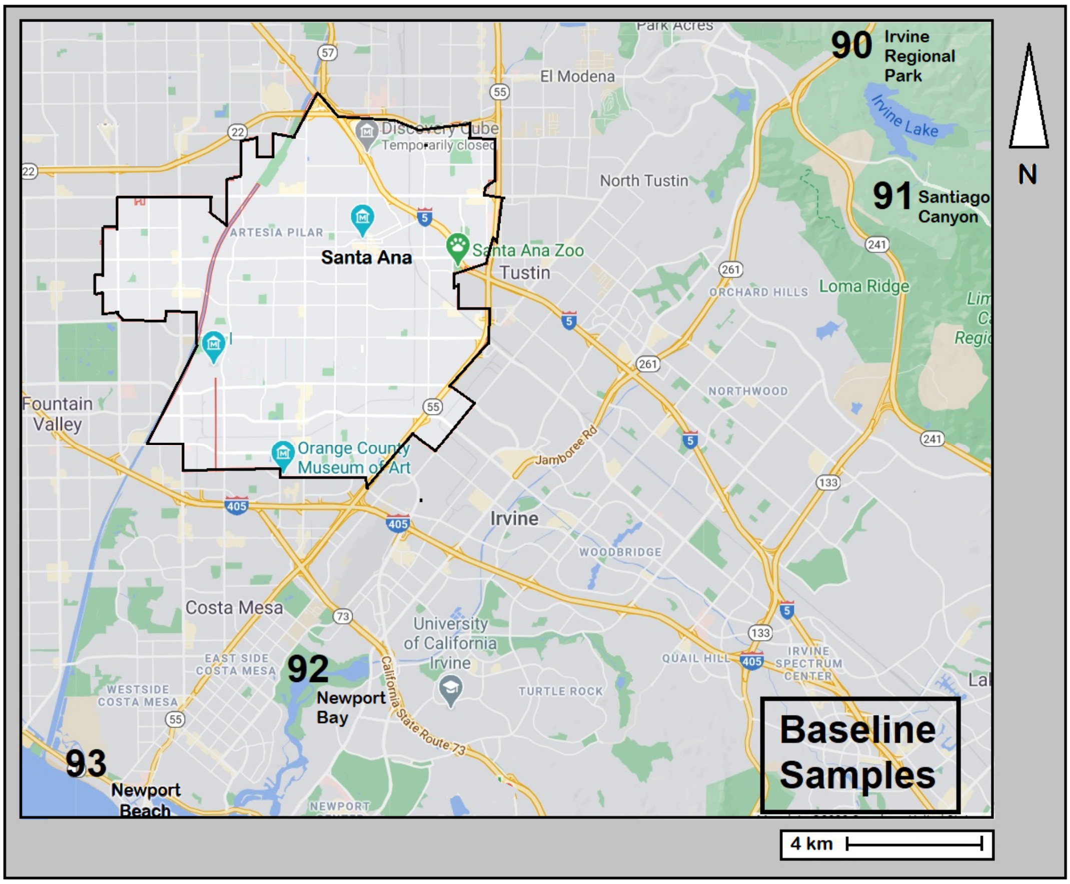

Figure S1. Locations of all four baseline sampling sites (#90-93) within Irvine Regional Park, Santiago Canyon, Newport Beach, and the Newport Bay relative to Santa Ana city boundary.

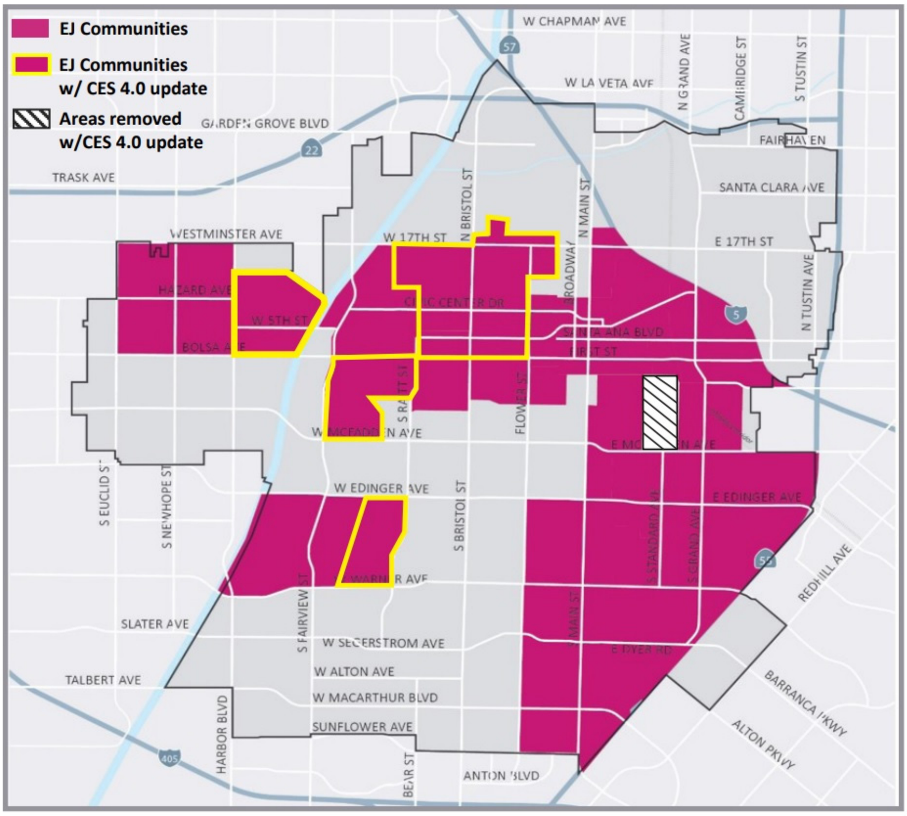

Figure S2. Locations of environmental justice communities within Santa Ana, CA, as of the 2021 updated designation.

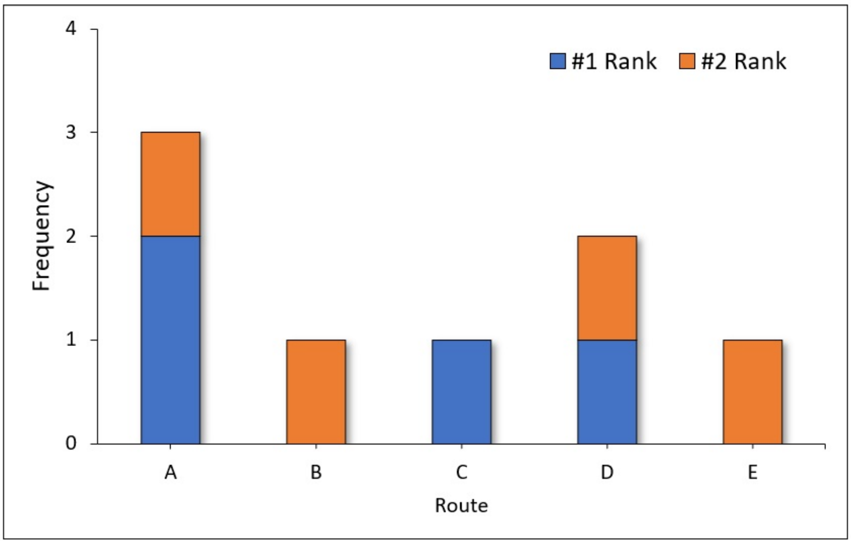

Figure S3. Number of times (frequency) that the monthly sampling day average $PM_{2.5}$ concentration within a route was ranked either highest (#1 rank) or second highest (#2 rank) when compared to the other routes across the four sampling days.

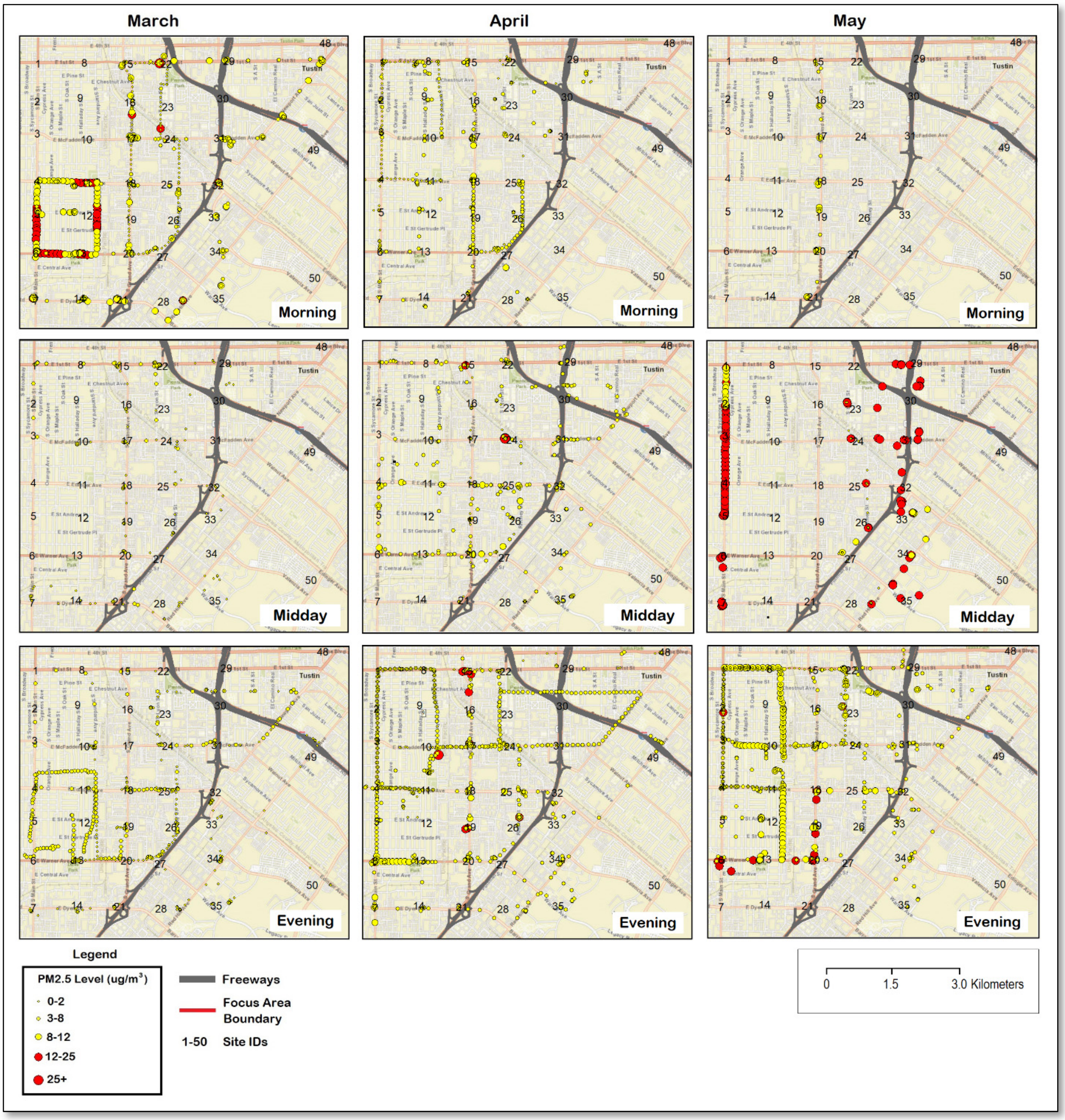

Figure S4. One-minute average $PM_{2.5}$ measurements projected across the Focus Area in Santa Ana, broken down by morning, afternoon, and evening measurements for each monthly sampling day. A failed GPS device led to missing GPS coordinates in some areas for March and May.
